# Supplementary material for: Cotranslational folding of alkaline phosphatase in the periplasm of Escherichia coli
Source: Protein Sci. 2020 Aug 24;29(10):2028–37. doi: 10.1002/pro.3927 (PMC7513700; doi:10.1002/pro.3927)
Supplement: Supplementary file 1 — APPENDIX S1: Supporting Information [file PRO-29-2028-s001.zip › PRO_3927_Supplementary information.pdf]

Supplementary Material 1

Sequences of the NC PhoA constructs

Key: PhoA NC Signal Sequence – PhoA mature domain – SGSG – Linker extension – HA tag – SecM AP – C-terminal tail

Name	Sequence
N= 555	MKQSTIALALLPLLFTPVTKPRTPEMPVLENRAAQGDITAPGGARRLTG<br>DQTAALRDSLSDKPAKNIILLIGDGMGDSEITAARNYAEGAGGFFKGID<br>ALPLTGQYTHYALNKKTGKPDYVTDSAASATAWSTGVKTYNGALGVDIH<br>EKDHPTILEMAKAAGLATGNVSTAELQDATPAALVAHVTSRKCYGPSAT<br>SEKCPGNALEKGGKGSITEQLLNARADVTLGGGAKTFAETATAGEWQGK<br>TLREQAQARGYQLVSDAASLNSVTEANQQKPLLGLFADGNMPVRWLGPK<br>ATYHGNIDKPAVTCTPNPQRNDSVPTLAQMTDKAIELLSKNEKGFFLQV<br>EGASIDKQDHAANPCGQIGETVDLDEAVQRALEFAKKEGNTLVIVTADH<br>AHASQIVAPDTKAPGLTQALNTKDGAVMVMSYGNSEEDSQEHTGSQLRI<br>AAYGPHAANVVGLTDQTDLFYTMKAALGLKSGSGSGSGYPYDVPDYAMY<br>PYDVPDYAMYPYDVPDYAMYPYDVPDYAMYPYDVPDYAMYPYDVPDYAM<br>YFSTPVWISQAQGIRAGPGSSDKQEGEWPTGLRLSRIGGIHSLAVVLQR<br>RDWENPGVTQLNRLAAHPPFASWRNSEEARTDRPSQQLRSLNGEWR*
N= 501	MKQSTIALALLPLLFTPVTKPRMKQSTIALALLPLLFTPVTKPRTPEMP<br>VLENRAAQGDITAPGGARRLTGDQTAALRDSLSDKPAKNIILLIGDGMG<br>DSEITAARNYAEGAGGFFKGIDALPLTGQYTHYALNKKTGKPDYVTDSA<br>ASATAWSTGVKTYNGALGVDIHEKDHPTILEMAKAAGLATGNVSTAELQ<br>DATPAALVAHVTSRKCYGPSATSEKCPGNALEKGGKGSITEQLLNARAD<br>VTLGGGAKTFAETATAGEWQGKTLREQAQARGYQLVSDAASLNSVTEAN<br>QQKPLLGLFADGNMPVRWLGPKATYHGNIDKPAVTCTPNPQRNDSVPTL<br>AQMTDKAIELLSKNEKGFFLQVEGASIDKQDHAANPCGQIGETVDLDEA<br>VQRALEFAKKEGNTLVIVTADHAHASQIVAPDTKAPGLTQALNTKDGAV<br>MVMSYGNSEEDSQEHTGSQLRIAAYGPHAANVVGLTDQTDLFYTMKAAL<br>GLKSGSGYPYDVPDYAFSTPVWISQAQGIRAGPGSSDKQEGEWPTGLRL<br>SRIGGIHSLAVVLQRRDWENPGVTQLNRLAAHPPFASWRNSEEARTDRP<br>SQQLRSLNGEWR*
N= 491	MKQSTIALALLPLLFTPVTKPRMKQSTIALALLPLLFTPVTKPRTPEMP<br>VLENRAAQGDITAPGGARRLTGDQTAALRDSLSDKPAKNIILLIGDGMG<br>DSEITAARNYAEGAGGFFKGIDALPLTGQYTHYALNKKTGKPDYVTDSA<br>ASATAWSTGVKTYNGALGVDIHEKDHPTILEMAKAAGLATGNVSTAELQ<br>DATPAALVAHVTSRKCYGPSATSEKCPGNALEKGGKGSITEQLLNARAD<br>VTLGGGAKTFAETATAGEWQGKTLREQAQARGYQLVSDAASLNSVTEAN<br>QQKPLLGLFADGNMPVRWLGPKATYHGNIDKPAVTCTPNPQRNDSVPTL<br>AQMTDKAIELLSKNEKGFFLQVEGASIDKQDHAANPCGQIGETVDLDEA<br>VQRALEFAKKEGNTLVIVTADHAHASQIVAPDTKAPGLTQALNTKDGAV<br>MVMSYGNSEEDSQEHTGSQLRIAAYGPHAANVVGLTDQTDLFSGSGYPY<br>DVPDYAFSTPVWISQAQGIRAGPGSSDKQEGEWPTGLRLSRIGGIHSLA<br>VVLQRRDWENPGVTQLNRLAAHPPFASWRNSEEARTDRPSQQLRSLNGE<br>WR*
N= 471	MKQSTIALALLPLLFTPVTKPRMKQSTIALALLPLLFTPVTKPRTPEMP<br>VLENRAAQGDITAPGGARRLTGDQTAALRDSLSDKPAKNIILLIGDGMG<br>DSEITAARNYAEGAGGFFKGIDALPLTGQYTHYALNKKTGKPDYVTDSA<br>ASATAWSTGVKTYNGALGVDIHEKDHPTILEMAKAAGLATGNVSTAELQ<br>DATPAALVAHVTSRKCYGPSATSEKCPGNALEKGGKGSITEQLLNARAD<br>VTLGGGAKTFAETATAGEWQGKTLREQAQARGYQLVSDAASLNSVTEAN

	QQKPLLGLFADGNMPVRWLGPKATYHGNIDKPAVTCTPNPQRNDSVPTL AQMTDKAIELLSKNEKGFFLQVEGASIDKQDHAANPCGQIGETVDLDEA VQRALEFAKKEGNTLVIVTADHAHASQIVAPDTKAPGLTQALNTKDGAV MVMSYGNSEEDSQEHTGSQLRISGSGYPYDVPDYAFSTPVWISQAQGIR AGPGSSDKQEGEWPTGLRLSRIGGIHSLAVVLQRRDWENPGVTQLNRLA AHPPFASWRNSEEARTDRPSQQLRSLNGEWR*
N= 461	MKQSTIALALLPLLFTPVTKPRMKQSTIALALLPLLFTPVTKPRTPEMP VLENRAAQGDITAPGGARRLTGDQTAALRDSLSDKPAKNIILLIGDGMG DSEITAARNYAEGAGGFFKGIDALPLTGQYTHYALNKKTGKPDYVTDSA ASATAWSTGVKTYNGALGVDIHEKDHPTILEMAKAAGLATGNVSTAELQ DATPAALVAHVTSRKCYGPSATSEKCPGNALEKGGKGSITEQLLNARAD VTLGGGAKTFAETATAGEWQGKTLREQAQARGYQLVSDAASLNSVTEAN QQKPLLGLFADGNMPVRWLGPKATYHGNIDKPAVTCTPNPQRNDSVPTL AQMTDKAIELLSKNEKGFFLQVEGASIDKQDHAANPCGQIGETVDLDEA VQRALEFAKKEGNTLVIVTADHAHASQIVAPDTKAPGLTQALNTKDGAV MVMSYGNSEEDSSGSGYPYDVPDYAFSTPVWISQAQGIRAGPGSSDKQE GEWPTGLRLSRIGGIHSLAVVLQRRDWENPGVTQLNRLAAHPPFASWRN SEEARTDRPSQQLRSLNGEWR*
N= 450	MKQSTIALALLPLLFTPVTKPRMKQSTIALALLPLLFTPVTKPRTPEMP VLENRAAQGDITAPGGARRLTGDQTAALRDSLSDKPAKNIILLIGDGMG DSEITAARNYAEGAGGFFKGIDALPLTGQYTHYALNKKTGKPDYVTDSA ASATAWSTGVKTYNGALGVDIHEKDHPTILEMAKAAGLATGNVSTAELQ DATPAALVAHVTSRKCYGPSATSEKCPGNALEKGGKGSITEQLLNARAD VTLGGGAKTFAETATAGEWQGKTLREQAQARGYQLVSDAASLNSVTEAN QQKPLLGLFADGNMPVRWLGPKATYHGNIDKPAVTCTPNPQRNDSVPTL AQMTDKAIELLSKNEKGFFLQVEGASIDKQDHAANPCGQIGETVDLDEA VQRALEFAKKEGNTLVIVTADHAHASQIVAPDTKAPGLTQALNTKDGAV MSGSGYPYDVPDYAFSTPVWISQAQGIRAGPGSSDKQEGEWPTGLRLSR IGGIHSLAVVLQRRDWENPGVTQLNRLAAHPPFASWRNSEEARTDRPSQ QLRSLNGEWR*
N= 440	MKQSTIALALLPLLFTPVTKPRMKQSTIALALLPLLFTPVTKPRTPEMP VLENRAAQGDITAPGGARRLTGDQTAALRDSLSDKPAKNIILLIGDGMG DSEITAARNYAEGAGGFFKGIDALPLTGQYTHYALNKKTGKPDYVTDSA ASATAWSTGVKTYNGALGVDIHEKDHPTILEMAKAAGLATGNVSTAELQ DATPAALVAHVTSRKCYGPSATSEKCPGNALEKGGKGSITEQLLNARAD VTLGGGAKTFAETATAGEWQGKTLREQAQARGYQLVSDAASLNSVTEAN QQKPLLGLFADGNMPVRWLGPKATYHGNIDKPAVTCTPNPQRNDSVPTL AQMTDKAIELLSKNEKGFFLQVEGASIDKQDHAANPCGQIGETVDLDEA VQRALEFAKKEGNTLVIVTADHAHASQIVAPDTKAPGLTQSGSGYPYDV PDYAFSTPVWISQAQGIRAGPGSSDKQEGEWPTGLRLSRIGGIHSLAVV LQRRDWENPGVTQLNRLAAHPPFASWRNSEEARTDRPSQQLRSLNGEWR*
N= 435	MKQSTIALALLPLLFTPVTKPRMKQSTIALALLPLLFTPVTKPRTPEMP VLENRAAQGDITAPGGARRLTGDQTAALRDSLSDKPAKNIILLIGDGMG DSEITAARNYAEGAGGFFKGIDALPLTGQYTHYALNKKTGKPDYVTDSA ASATAWSTGVKTYNGALGVDIHEKDHPTILEMAKAAGLATGNVSTAELQ DATPAALVAHVTSRKCYGPSATSEKCPGNALEKGGKGSITEQLLNARAD VTLGGGAKTFAETATAGEWQGKTLREQAQARGYQLVSDAASLNSVTEAN QQKPLLGLFADGNMPVRWLGPKATYHGNIDKPAVTCTPNPQRNDSVPTL AQMTDKAIELLSKNEKGFFLQVEGASIDKQDHAANPCGQIGETVDLDEA VQRALEFAKKEGNTLVIVTADHAHASQIVAPDTKASGSGYPYDVPDYAF STPVWISQAQGIRAGPGSSDKQEGEWPTGLRLSRIGGIHSLAVVLQRRD WENPGVTQLNRLAAHPPFASWRNSEEARTDRPSQQLRSLNGEWR*

N= 431	MKQSTIALALLPLLFTPVTKPRMKQSTIALALLPLLFTPVTKPRTPEMP VLENRAAQGDITAPGGARRLTGDQTAALRDSLSDKPAKNIILLIGDGMG DSEITAARNYAEGAGGFFKGIDALPLTGQYTHYALNKKTGKPDYVTDSA ASATAWSTGVKTYNGALGVDIHEKDHPTILEMAKAAGLATGNVSTAELQ DATPAALVAHVTSRKCYGPSATSEKCPGNALEKGGKGSITEQLLNARAD VTLGGGAKTFAETATAGEWQGKTLREQAQARGYQLVSDAASLNSVTEAN QQKPLLGLFADGNMPVRWLGPKATYHGNIDKPAVTCTPNPQRNDSVPTL AQMTDKAIELLSKNEKGFFLQVEGASIDKQDHAANPCGQIGETVDLDEA VQRALEFAKKEGNTLVIVTADHAHASQIVAPSGSGYPYDVPDYAFSTPV WISQAQGIRAGPGSSDKQEGEWPTGLRLSRIGGIHSLAVVLQRRDWENP GVTQLNRLAAHPPFASWRNSEEARTDRPSQQLRSLNGEWR*
N= 426	MKQSTIALALLPLLFTPVTKPRMKQSTIALALLPLLFTPVTKPRTPEMP VLENRAAQGDITAPGGARRLTGDQTAALRDSLSDKPAKNIILLIGDGMG DSEITAARNYAEGAGGFFKGIDALPLTGQYTHYALNKKTGKPDYVTDSA ASATAWSTGVKTYNGALGVDIHEKDHPTILEMAKAAGLATGNVSTAELQ DATPAALVAHVTSRKCYGPSATSEKCPGNALEKGGKGSITEQLLNARAD VTLGGGAKTFAETATAGEWQGKTLREQAQARGYQLVSDAASLNSVTEAN QQKPLLGLFADGNMPVRWLGPKATYHGNIDKPAVTCTPNPQRNDSVPTL AQMTDKAIELLSKNEKGFFLQVEGASIDKQDHAANPCGQIGETVDLDEA VQRALEFAKKEGNTLVIVTADHAHASSGSGYPYDVPDYAFSTPVWISQA QGIRAGPGSSDKQEGEWPTGLRLSRIGGIHSLAVVLQRRDWENPGVTQL NRLAAHPPFASWRNSEEARTDRPSQQLRSLNGEWR*
N= 421	MKQSTIALALLPLLFTPVTKPRMKQSTIALALLPLLFTPVTKPRTPEMP VLENRAAQGDITAPGGARRLTGDQTAALRDSLSDKPAKNIILLIGDGMG DSEITAARNYAEGAGGFFKGIDALPLTGQYTHYALNKKTGKPDYVTDSA ASATAWSTGVKTYNGALGVDIHEKDHPTILEMAKAAGLATGNVSTAELQ DATPAALVAHVTSRKCYGPSATSEKCPGNALEKGGKGSITEQLLNARAD VTLGGGAKTFAETATAGEWQGKTLREQAQARGYQLVSDAASLNSVTEAN QQKPLLGLFADGNMPVRWLGPKATYHGNIDKPAVTCTPNPQRNDSVPTL AQMTDKAIELLSKNEKGFFLQVEGASIDKQDHAANPCGQIGETVDLDEA VQRALEFAKKEGNTLVIVTADSGSGYPYDVPDYAFSTPVWISQAQGIRA GPGSSDKQEGEWPTGLRLSRIGGIHSLAVVLQRRDWENPGVTQLNRLAA HPPFASWRNSEEARTDRPSQQLRSLNGEWR*
N= 415	MKQSTIALALLPLLFTPVTKPRMKQSTIALALLPLLFTPVTKPRTPEMP VLENRAAQGDITAPGGARRLTGDQTAALRDSLSDKPAKNIILLIGDGMG DSEITAARNYAEGAGGFFKGIDALPLTGQYTHYALNKKTGKPDYVTDSA ASATAWSTGVKTYNGALGVDIHEKDHPTILEMAKAAGLATGNVSTAELQ DATPAALVAHVTSRKCYGPSATSEKCPGNALEKGGKGSITEQLLNARAD VTLGGGAKTFAETATAGEWQGKTLREQAQARGYQLVSDAASLNSVTEAN QQKPLLGLFADGNMPVRWLGPKATYHGNIDKPAVTCTPNPQRNDSVPTL AQMTDKAIELLSKNEKGFFLQVEGASIDKQDHAANPCGQIGETVDLDEA VQRALEFAKKEGNTLSGSGYPYDVPDYAFSTPVWISQAQGIRAGPGSSD KQEGEWPTGLRLSRIGGIHSLAVVLQRRDWENPGVTQLNRLAAHPPFAS WRNSEEARTDRPSQQLRSLNGEWR*
N= 411	MKQSTIALALLPLLFTPVTKPRMKQSTIALALLPLLFTPVTKPRTPEMP VLENRAAQGDITAPGGARRLTGDQTAALRDSLSDKPAKNIILLIGDGMG DSEITAARNYAEGAGGFFKGIDALPLTGQYTHYALNKKTGKPDYVTDSA ASATAWSTGVKTYNGALGVDIHEKDHPTILEMAKAAGLATGNVSTAELQ DATPAALVAHVTSRKCYGPSATSEKCPGNALEKGGKGSITEQLLNARAD VTLGGGAKTFAETATAGEWQGKTLREQAQARGYQLVSDAASLNSVTEAN QQKPLLGLFADGNMPVRWLGPKATYHGNIDKPAVTCTPNPQRNDSVPTL AQMTDKAIELLSKNEKGFFLQVEGASIDKQDHAANPCGQIGETVDLDEA

	VQRALEFAKKESGSGYPYDVPDYAFSTPVWISQAQGIRAGPGSSDKQEG<br>EWPTGLRLSRIGGIHSLAVVLQRRDWENPGVTQLNRLAAHPPFASWRNS<br>EEARTDRPSQQLRSLNGEWR*
N= 409	MKQSTIALALLPLLFTPVTKPRMKQSTIALALLPLLFTPVTKPRTPEMP<br>VLENRAAQGDITAPGGARRLTGDQTAALRDSLSDKPAKNIILLIGDGMG<br>DSEITAARNYAEGAGGFFKGIDALPLTGQYTHYALNKKTGKPDYVTDSA<br>ASATAWSTGVKTYNGALGVDIHEKDHPTILEMAKAAGLATGNVSTAELQ<br>DATPAALVAHVTSRKCYGPSATSEKCPGNALEKGGKGSITEQLLNARAD<br>VTLGGGAKTFAETATAGEWQGKTLREQAQARGYQLVSDAASLNSVTEAN<br>QQKPLLGLFADGNMPVRWLGPKATYHGNIDKPAVTCTPNPQRNDSVPTL<br>AQMTDKAIELLSKNEKGFFLQVEGASIDKQDHAANPCGQIGETVDLDEA<br>VQRALEFAKSGSGYPYDVPDYAFSTPVWISQAQGIRAGPGSSDKQEGEW<br>PTGLRLSRIGGIHSLAVVLQRRDWENPGVTQLNRLAAHPPFASWRNS<br>EEARTDRPSQQLRSLNGEWR*
N= 407	MKQSTIALALLPLLFTPVTKPRMKQSTIALALLPLLFTPVTKPRTPEMP<br>VLENRAAQGDITAPGGARRLTGDQTAALRDSLSDKPAKNIILLIGDGMG<br>DSEITAARNYAEGAGGFFKGIDALPLTGQYTHYALNKKTGKPDYVTDSA<br>ASATAWSTGVKTYNGALGVDIHEKDHPTILEMAKAAGLATGNVSTAELQ<br>DATPAALVAHVTSRKCYGPSATSEKCPGNALEKGGKGSITEQLLNARAD<br>VTLGGGAKTFAETATAGEWQGKTLREQAQARGYQLVSDAASLNSVTEAN<br>QQKPLLGLFADGNMPVRWLGPKATYHGNIDKPAVTCTPNPQRNDSVPTL<br>AQMTDKAIELLSKNEKGFFLQVEGASIDKQDHAANPCGQIGETVDLDEA<br>VQRALEFSGSGYPYDVPDYAFSTPVWISQAQGIRAGPGSSDKQEGEWPT<br>GLRLSRIGGIHSLAVVLQRRDWENPGVTQLNRLAAHPPFASWRNSEEAR<br>TDRPSQQLRSLNGEWR*
N= 405	MKQSTIALALLPLLFTPVTKPRMKQSTIALALLPLLFTPVTKPRTPEMP<br>VLENRAAQGDITAPGGARRLTGDQTAALRDSLSDKPAKNIILLIGDGMG<br>DSEITAARNYAEGAGGFFKGIDALPLTGQYTHYALNKKTGKPDYVTDSA<br>ASATAWSTGVKTYNGALGVDIHEKDHPTILEMAKAAGLATGNVSTAELQ<br>DATPAALVAHVTSRKCYGPSATSEKCPGNALEKGGKGSITEQLLNARAD<br>VTLGGGAKTFAETATAGEWQGKTLREQAQARGYQLVSDAASLNSVTEAN<br>QQKPLLGLFADGNMPVRWLGPKATYHGNIDKPAVTCTPNPQRNDSVPTL<br>AQMTDKAIELLSKNEKGFFLQVEGASIDKQDHAANPCGQIGETVDLDEA<br>VQRALSGSGYPYDVPDYAFSTPVWISQAQGIRAGPGSSDKQEGEWPTGL<br>RLSRIGGIHSLAVVLQRRDWENPGVTQLNRLAAHPPFASWRNSEEARTD<br>RPSQQLRSLNGEWR*
N= 403	MKQSTIALALLPLLFTPVTKPRMKQSTIALALLPLLFTPVTKPRTPEMP<br>VLENRAAQGDITAPGGARRLTGDQTAALRDSLSDKPAKNIILLIGDGMG<br>DSEITAARNYAEGAGGFFKGIDALPLTGQYTHYALNKKTGKPDYVTDSA<br>ASATAWSTGVKTYNGALGVDIHEKDHPTILEMAKAAGLATGNVSTAELQ<br>DATPAALVAHVTSRKCYGPSATSEKCPGNALEKGGKGSITEQLLNARAD<br>VTLGGGAKTFAETATAGEWQGKTLREQAQARGYQLVSDAASLNSVTEAN<br>QQKPLLGLFADGNMPVRWLGPKATYHGNIDKPAVTCTPNPQRNDSVPTL<br>AQMTDKAIELLSKNEKGFFLQVEGASIDKQDHAANPCGQIGETVDLDEA<br>VQRSGSGYPYDVPDYAFSTPVWISQAQGIRAGPGSSDKQEGEWPTGLRL<br>SRIGGIHSLAVVLQRRDWENPGVTQLNRLAAHPPFASWRNSEEARTDRP<br>SQQLRSLNGEWR*
N= 401	MKQSTIALALLPLLFTPVTKPRMKQSTIALALLPLLFTPVTKPRTPEMP<br>VLENRAAQGDITAPGGARRLTGDQTAALRDSLSDKPAKNIILLIGDGMG<br>DSEITAARNYAEGAGGFFKGIDALPLTGQYTHYALNKKTGKPDYVTDSA<br>ASATAWSTGVKTYNGALGVDIHEKDHPTILEMAKAAGLATGNVSTAELQ<br>DATPAALVAHVTSRKCYGPSATSEKCPGNALEKGGKGSITEQLLNARAD

	VTLGGGAKTFAETATAGEWQGKTLREQAQARGYQLVSDAASLNSVTEAN QQKPLLGLFADGNMPVRWLGPKATYHGNIDKPAVTCTPNPQRNDSVPTL AQMTDKAIELLSKNEKGFFLQVEGASIDKQDHAANPCGQIGETVDLDEA VSGSGYPYDVPDYAFSTPVWISQAQGIRAGPGSSDKQEGEWPTGLRLSR IGGIHSLAVVLQRRDWENPGVTQLNRLAAHPPFASWRNSEEARTDRPS QQLRSLNGEWR*
N= 391	MKQSTIALALLPLLFTPVTKPRMKQSTIALALLPLLFTPVTKPRTPEMP VLENRAAQGDITAPGGARRLTGDQTAALRDSLSDKPAKNIILLIGDGMG DSEITAARNYAEGAGGFFKGIDALPLTGQYTHYALNKKTGKPDYVTDSA ASATAWSTGVKTYNGALGVDIHEKDHPTILEMAKAAGLATGNVSTAELQ DATPAALVAHVTSRKCYGPSATSEKCPGNALEKGGKGSITEQLLNARAD VTLGGGAKTFAETATAGEWQGKTLREQAQARGYQLVSDAASLNSVTEAN QQKPLLGLFADGNMPVRWLGPKATYHGNIDKPAVTCTPNPQRNDSVPTL AQMTDKAIELLSKNEKGFFLQVEGASIDKQDHAANPCGQISGSGYPYDV PDYAFSTPVWISQAQGIRAGPGSSDKQEGEWPTGLRLSRIGGIHSLAVV LQRRDWENPGVTQLNRLAAHPPFASWRNSEEARTDRPSQQLRSLNGEWR *
N= 381	MKQSTIALALLPLLFTPVTKPRMKQSTIALALLPLLFTPVTKPRTPEMP VLENRAAQGDITAPGGARRLTGDQTAALRDSLSDKPAKNIILLIGDGMG DSEITAARNYAEGAGGFFKGIDALPLTGQYTHYALNKKTGKPDYVTDSA ASATAWSTGVKTYNGALGVDIHEKDHPTILEMAKAAGLATGNVSTAELQ DATPAALVAHVTSRKCYGPSATSEKCPGNALEKGGKGSITEQLLNARAD VTLGGGAKTFAETATAGEWQGKTLREQAQARGYQLVSDAASLNSVTEAN QQKPLLGLFADGNMPVRWLGPKATYHGNIDKPAVTCTPNPQRNDSVPTL AQMTDKAIELLSKNEKGFFLQVEGASIDKQSGSGYPYDVPDYAFSTPVW ISQAQGIRAGPGSSDKQEGEWPTGLRLSRIGGIHSLAVVLQRRDWENPG VTQLNRLAAHPPFASWRNSEEARTDRPSQQLRSLNGEWR*
N= 371	MKQSTIALALLPLLFTPVTKPRMKQSTIALALLPLLFTPVTKPRTPEMP VLENRAAQGDITAPGGARRLTGDQTAALRDSLSDKPAKNIILLIGDGMG DSEITAARNYAEGAGGFFKGIDALPLTGQYTHYALNKKTGKPDYVTDSA ASATAWSTGVKTYNGALGVDIHEKDHPTILEMAKAAGLATGNVSTAELQ DATPAALVAHVTSRKCYGPSATSEKCPGNALEKGGKGSITEQLLNARAD VTLGGGAKTFAETATAGEWQGKTLREQAQARGYQLVSDAASLNSVTEAN QQKPLLGLFADGNMPVRWLGPKATYHGNIDKPAVTCTPNPQRNDSVPTL AQMTDKAIELLSKNEKGFFLSGSGYPYDVPDYAFSTPVWISQAQGIRAG PGSSDKQEGEWPTGLRLSRIGGIHSLAVVLQRRDWENPGVTQLNRLAAH PPFASWRNSEEARTDRPSQQLRSLNGEWR*
N= 361	MKQSTIALALLPLLFTPVTKPRMKQSTIALALLPLLFTPVTKPRTPEMP VLENRAAQGDITAPGGARRLTGDQTAALRDSLSDKPAKNIILLIGDGMG DSEITAARNYAEGAGGFFKGIDALPLTGQYTHYALNKKTGKPDYVTDSA ASATAWSTGVKTYNGALGVDIHEKDHPTILEMAKAAGLATGNVSTAELQ DATPAALVAHVTSRKCYGPSATSEKCPGNALEKGGKGSITEQLLNARAD VTLGGGAKTFAETATAGEWQGKTLREQAQARGYQLVSDAASLNSVTEAN QQKPLLGLFADGNMPVRWLGPKATYHGNIDKPAVTCTPNPQRNDSVPTL AQMTDKAIELSGSGYPYDVPDYAFSTPVWISQAQGIRAGPGSSDKQEGE WPTGLRLSRIGGIHSLAVVLQRRDWENPGVTQLNRLAAHPPFASWRNSE EARTDRPSQQLRSLNGEWR*
N= 351	MKQSTIALALLPLLFTPVTKPRMKQSTIALALLPLLFTPVTKPRTPEMP VLENRAAQGDITAPGGARRLTGDQTAALRDSLSDKPAKNIILLIGDGMG DSEITAARNYAEGAGGFFKGIDALPLTGQYTHYALNKKTGKPDYVTDSA ASATAWSTGVKTYNGALGVDIHEKDHPTILEMAKAAGLATGNVSTAELQ DATPAALVAHVTSRKCYGPSATSEKCPGNALEKGGKGSITEQLLNARAD

	VTLGGGAKTFAETATAGEWQGKTLREQAQARGYQLVSDAASLNSVTEAN<br>QQKPLLGLFADGNMPVRWLGPKATYHGNIDKPAVTCTPNPQRNDSVPTL<br>SGSGYPYDVPDYAFSTPVWISQAQGIRAGPGSSDKQEGEWPTGLRLSRI<br>GGIHSLAVVLQRRDWENPGVTQLNRLAAHPPFASWRNSEEARTDRPSQQ<br>LRSLNGEWR*
N= 341	MKQSTIALALLPLLFTPVTKPRMKQSTIALALLPLLFTPVTKPRTPEMP<br>VLENRAAQGDITAPGGARRLTGDQTAALRDSLSDKPAKNIILLIGDGMG<br>DSEITAARNYAEGAGGFFKGIDALPLTGQYTHYALNKKTGKPDYVTDSA<br>ASATAWSTGVKTYNGALGVDIHEKDHPTILEMAKAAGLATGNVSTAELQ<br>DATPAALVAHVTSRKCYGPSATSEKCPGNALEKGGKGSITEQLLNARAD<br>VTLGGGAKTFAETATAGEWQGKTLREQAQARGYQLVSDAASLNSVTEAN<br>QQKPLLGLFADGNMPVRWLGPKATYHGNIDKPAVTCTPNPSGSGYPYDV<br>PDYAFSTPVWISQAQGIRAGPGSSDKQEGEWPTGLRLSRIGGIHSLAVV<br>LQRRDWENPGVTQLNRLAAHPPFASWRNSEEARTDRPSQQLRSLNGEWR<br>*
N= 331	MKQSTIALALLPLLFTPVTKPRMKQSTIALALLPLLFTPVTKPRTPEMP<br>VLENRAAQGDITAPGGARRLTGDQTAALRDSLSDKPAKNIILLIGDGMG<br>DSEITAARNYAEGAGGFFKGIDALPLTGQYTHYALNKKTGKPDYVTDSA<br>ASATAWSTGVKTYNGALGVDIHEKDHPTILEMAKAAGLATGNVSTAELQ<br>DATPAALVAHVTSRKCYGPSATSEKCPGNALEKGGKGSITEQLLNARAD<br>VTLGGGAKTFAETATAGEWQGKTLREQAQARGYQLVSDAASLNSVTEAN<br>QQKPLLGLFADGNMPVRWLGPKATYHGNISGSGYPYDVPDYAFSTPVWI<br>SQAQGIRAGPGSSDKQEGEWPTGLRLSRIGGIHSLAVVLQRRDWENPGV<br>TQLNRLAAHPPFASWRNSEEARTDRPSQQLRSLNGEWR*
N= 321	MKQSTIALALLPLLFTPVTKPRMKQSTIALALLPLLFTPVTKPRTPEMP<br>VLENRAAQGDITAPGGARRLTGDQTAALRDSLSDKPAKNIILLIGDGMG<br>DSEITAARNYAEGAGGFFKGIDALPLTGQYTHYALNKKTGKPDYVTDSA<br>ASATAWSTGVKTYNGALGVDIHEKDHPTILEMAKAAGLATGNVSTAELQ<br>DATPAALVAHVTSRKCYGPSATSEKCPGNALEKGGKGSITEQLLNARAD<br>VTLGGGAKTFAETATAGEWQGKTLREQAQARGYQLVSDAASLNSVTEAN<br>QQKPLLGLFADGNMPVRWLSGSGYPYDVPDYAFSTPVWISQAQGIRAGP<br>GSSDKQEGEWPTGLRLSRIGGIHSLAVVLQRRDWENPGVTQLNRLAAHP<br>PFASWRNSEEARTDRPSQQLRSLNGEWR*
N= 311	MKQSTIALALLPLLFTPVTKPRMKQSTIALALLPLLFTPVTKPRTPEMP<br>VLENRAAQGDITAPGGARRLTGDQTAALRDSLSDKPAKNIILLIGDGMG<br>DSEITAARNYAEGAGGFFKGIDALPLTGQYTHYALNKKTGKPDYVTDSA<br>ASATAWSTGVKTYNGALGVDIHEKDHPTILEMAKAAGLATGNVSTAELQ<br>DATPAALVAHVTSRKCYGPSATSEKCPGNALEKGGKGSITEQLLNARAD<br>VTLGGGAKTFAETATAGEWQGKTLREQAQARGYQLVSDAASLNSVTEAN<br>QQKPLLGLFSGSGYPYDVPDYAFSTPVWISQAQGIRAGPGSSDKQEGEW<br>PTGLRLSRIGGIHSLAVVLQRRDWENPGVTQLNRLAAHPPFASWRNSEE<br>ARTDRPSQQLRSLNGEWR*
N= 307	MKQSTIALALLPLLFTPVTKPRMKQSTIALALLPLLFTPVTKPRTPEMP<br>VLENRAAQGDITAPGGARRLTGDQTAALRDSLSDKPAKNIILLIGDGMG<br>DSEITAARNYAEGAGGFFKGIDALPLTGQYTHYALNKKTGKPDYVTDSA<br>ASATAWSTGVKTYNGALGVDIHEKDHPTILEMAKAAGLATGNVSTAELQ<br>DATPAALVAHVTSRKCYGPSATSEKCPGNALEKGGKGSITEQLLNARAD<br>VTLGGGAKTFAETATAGEWQGKTLREQAQARGYQLVSDAASLNSVTEAN<br>QQKPLSGSGYPYDVPDYAFSTPVWISQAQGIRAGPGSSDKQEGEWPTGL<br>RLSRIGGIHSLAVVLQRRDWENPGVTQLNRLAAHPPFASWRNSEEARTD<br>RPSQQLRSLNGEWR*
N= 305	MKQSTIALALLPLLFTPVTKPRMKQSTIALALLPLLFTPVTKPRTPEMP

	VLENRAAQGDITAPGGARRLTGDQTAALRDSLSDKPAKNIILLIGDGMG DSEITAARNYAEGAGGFFKGIDALPLTGQYTHYALNKKTGKPDYVTDSA ASATAWSTGVKTYNGALGVDIHEKDHPTILEMAKAAGLATGNVSTAELQ DATPAALVAHVTSRKCYGPSATSEKCPGNALEKGGKGSITEQLLNARAD VTLGGGAKTFAETATAGEWQGKTLREQAQARGYQLVSDAASLNSVTEAN QQKSGSGYPYDVPDYAFSTPVWISQAQGIRAGPGSSDKQEGEWPTGLRL SRIGGIHSLAVVLQRRDWENPGVTQLNRLAAHPPFASWRNSEEARTDRP SQQLRSLNGEWR*
N= 301	MKQSTIALALLPLLFTPVTKPRMKQSTIALALLPLLFTPVTKPRTPEMP VLENRAAQGDITAPGGARRLTGDQTAALRDSLSDKPAKNIILLIGDGMG DSEITAARNYAEGAGGFFKGIDALPLTGQYTHYALNKKTGKPDYVTDSA ASATAWSTGVKTYNGALGVDIHEKDHPTILEMAKAAGLATGNVSTAELQ DATPAALVAHVTSRKCYGPSATSEKCPGNALEKGGKGSITEQLLNARAD VTLGGGAKTFAETATAGEWQGKTLREQAQARGYQLVSDAASLNSVTEAS GSGYPYDVPDYAFSTPVWISQAQGIRAGPGSSDKQEGEWPTGLRLSRIG GIHSLAVVLQRRDWENPGVTQLNRLAAHPPFASWRNSEEARTDRPSQQL RSLNGEWR*
N= 291	MKQSTIALALLPLLFTPVTKPRMKQSTIALALLPLLFTPVTKPRTPEMP VLENRAAQGDITAPGGARRLTGDQTAALRDSLSDKPAKNIILLIGDGMG DSEITAARNYAEGAGGFFKGIDALPLTGQYTHYALNKKTGKPDYVTDSA ASATAWSTGVKTYNGALGVDIHEKDHPTILEMAKAAGLATGNVSTAELQ DATPAALVAHVTSRKCYGPSATSEKCPGNALEKGGKGSITEQLLNARAD VTLGGGAKTFAETATAGEWQGKTLREQAQARGYQLVSDSGSGYPYDVPD YAFSTPVWISQAQGIRAGPGSSDKQEGEWPTGLRLSRIGGIHSLAVVLQ RRDWENPGVTQLNRLAAHPPFASWRNSEEARTDRPSQQLRSLNGEWR*
N= 281	MKQSTIALALLPLLFTPVTKPRMKQSTIALALLPLLFTPVTKPRTPEMP VLENRAAQGDITAPGGARRLTGDQTAALRDSLSDKPAKNIILLIGDGMG DSEITAARNYAEGAGGFFKGIDALPLTGQYTHYALNKKTGKPDYVTDSA ASATAWSTGVKTYNGALGVDIHEKDHPTILEMAKAAGLATGNVSTAELQ DATPAALVAHVTSRKCYGPSATSEKCPGNALEKGGKGSITEQLLNARAD VTLGGGAKTFAETATAGEWQGKTLREQAQASGSGYPYDVPDYAFSTPVW ISQAQGIRAGPGSSDKQEGEWPTGLRLSRIGGIHSLAVVLQRRDWENPG VTQLNRLAAHPPFASWRNSEEARTDRPSQQLRSLNGEWR*
N= 271	MKQSTIALALLPLLFTPVTKPRMKQSTIALALLPLLFTPVTKPRTPEMP VLENRAAQGDITAPGGARRLTGDQTAALRDSLSDKPAKNIILLIGDGMG DSEITAARNYAEGAGGFFKGIDALPLTGQYTHYALNKKTGKPDYVTDSA ASATAWSTGVKTYNGALGVDIHEKDHPTILEMAKAAGLATGNVSTAELQ DATPAALVAHVTSRKCYGPSATSEKCPGNALEKGGKGSITEQLLNARAD VTLGGGAKTFAETATAGESGSGYPYDVPDYAFSTPVWISQAQGIRAGPG SSDKQEGEWPTGLRLSRIGGIHSLAVVLQRRDWENPGVTQLNRLAAHPP FASWRNSEEARTDRPSQQLRSLNGEWR*
N= 251	MKQSTIALALLPLLFTPVTKPRMKQSTIALALLPLLFTPVTKPRTPEMP VLENRAAQGDITAPGGARRLTGDQTAALRDSLSDKPAKNIILLIGDGMG DSEITAARNYAEGAGGFFKGIDALPLTGQYTHYALNKKTGKPDYVTDSA ASATAWSTGVKTYNGALGVDIHEKDHPTILEMAKAAGLATGNVSTAELQ DATPAALVAHVTSRKCYGPSATSEKCPGNALEKGGKGSITEQLLNARSG SGYPYDVPDYAFSTPVWISQAQGIRAGPGSSDKQEGEWPTGLRLSRIGG IHSLAVVLQRRDWENPGVTQLNRLAAHPPFASWRNSEEARTDRPSQQLR SLNGEWR*
N= 241	MKQSTIALALLPLLFTPVTKPRMKQSTIALALLPLLFTPVTKPRTPEMP VLENRAAQGDITAPGGARRLTGDQTAALRDSLSDKPAKNIILLIGDGMG DSEITAARNYAEGAGGFFKGIDALPLTGQYTHYALNKKTGKPDYVTDSA

	ASATAWSTGVKTYNGALGVDIHEKDHPTILEMAKAAGLATGNVSTAELQ<br>DATPAALVAHVTSRKCYGPSATSEKCPGNALEKGGKGSGSGYPYDVPDY<br>AFSTPVWISQAQGIRAGPGSSDKQEGEWPTGLRLSRIGGIHSLAVVLQR<br>RDWENPGVTQLNRLAAHPPFASWRNSEEARTDRPSQQLRSLNGEWR*
N= 141	MKQSTIALALLPLLFTPVTKPRMKQSTIALALLPLLFTPVTKPRTPEMP<br>VLENRAAQGDITAPGGARRLTGDQTAALRDSLSDKPAKNIILLIGDGMG<br>DSEITAARNYAEGAGGFFKGIDALPLTGQYTHYALSGSGYPYDVPDYAF<br>STPVWISQAQGIRAGPGSSDKQEGEWPTGLRLSRIGGIHSLAVVLQRRD<br>WENPGVTQLNRLAAHPPFASWRNSEEARTDRPSQQLRSLNGEWR*

Supplementary Material 2

List of Primers

Construct Name	Forward Primer 5’ → 3’	Reverse Primer 5’ → 3’
N= 491	TCAGGATCGGGCTACCC	TAGCCCGATCCTGAGAAGAGATCGG TCTGGTCG
N= 471	TCAGGATCGGGCTACCC	TAGCCCGATCCTGAAATACGCAACT GACTGCCG
N= 461	TCAGGATCGGGCTACCC	TAGCCCGATCCTGATGAATCCTCTT CGGAGTTCCC
N= 450	TCAGGATCGGGCTACCCATACG	CATCACTGCGCCATCTTTGGTATTT AGC
N= 440	TCAGGATCGGGCTACCCATACG	CTGGGTGAGGCCCGGAG
N= 435	TCAGGATCGGGCTACCCATACG	AGCTTTGGTATCCGGCGCAAC
N= 431	TCAGGATCGGGCTACCC	TAGCCCGATCCTGACGGCGCAACAA TCTGG
N= 426	TCAGGATCGGGCTACCCATACG	GCTGGCGTGGGCGTGATC
N= 421	TCAGGATCGGGCTACCC	TAGCCCGATCCTGAATCAGCGGTGA CTATGACCAG
N= 415	TCAGGATCGGGCTACCCATACG	CAGCGTGTTACCCTCCTTTTTAGC
N= 411	TCAGGATCGGGCTACCC	TAGCCCGATCCTGACTCCTTTTTAG CGAATTCCAGCG
N= 409	TCAGGATCGGGCTACCCATACG	TTTAGCGAATTCCAGCGCCCG
N= 407	TCAGGATCGGGCTACCCATACG	GAATTCCAGCGCCCGTTGTACG
N= 403	TCAGGATCGGGCTACCCATACG	CCGTTGTACGGCTTCATCGAGATC
N= 401	TCAGGATCGGGCTACCC	TAGCCCGATCCTGATACGGCTTCAT CGAGATCGAC
N= 391	TCAGGATCGGGCTACCC	TAGCCCGATCCTGACTGTTTATCGA TTGACGCACCTTC
N= 381	TCAGGATCGGGCTACCC	TAGCCCGATCCTGACAGGAAAAAGC CTTTCTCATTTTTACTCAAC
N= 371	TCAGGATCGGGCTACCC	TAGCCCGATCCTGACAATTCAATGG CTTTGTCGGTC
N= 361	TCAGGATCGGGCTACCC	TAGCCCGATCCTGACAGGGTTGGTA CACTGTCATTAC
N= 351	TCAGGATCGGGCTACCC	TAGCCCGATCCTGAATTTGGCGTAC AGGTGACTG
N= 341	TCAGGATCGGGCTACCC	TAGCCCGATCCTGAGATATTGCCAT GGTACGTTGCTTTC
N= 331	TCAGGATCGGGCTACCC	TAGCCCGATCCTGATAGCCAGCGCA CTGG
N= 321	TCAGGATCGGGCTACCC	TAGCCCGATCCTGAAAACAGGCCAA GCAGGGG
N= 311	TCAGGATCGGGCTACCC	TAGCCCGATCCTGATAGCCAGCGCA CTGG
N= 307	TCAGGATCGGGCTACCCATACG	CAGGGGTTTTTGCTGATTCGCTTC
N= 301	TCAGGATCGGGCTACCC	TAGCCCGATCCTGAATCGCTCACCA ACTGATAACC

N= 291	TCAGGATCGGGCTACCC	TAGCCCGATCCTGATGCCTGTTCACGC
N= 281	TCAGGATCGGGCTACCC	TAGCCCGATCCTGATTCACCAGCGGTTGCC
N= 271	TCAGGATCGGGCTACCC	TAGCCCGATCCTGATTTTGCGCCG
N= 251	TCAGGATCGGGCTACCC	TAGCCCGATCCTGATCCTTTTCCGCCTTTTTCCAG
N= 241	TCAGGATCGGGCTACCC	TAGCCCGATCCTGACGGACATTTTTCACTGGTCGCGCTCG
N= 231	TCAGGATCGGGCTACCC	TAGCCCGATCCTGAGTAGCATTTGCGCGAGG
N= 141	TCAGGATCGGGCTACCC	TAGCCCGATCCTGACAGCGCATAGTGAGTGTATTGC
C190A	GCATACGGTCCGAGCGCGA	TTTGCGCGAGGTCACATGTGC
C200A	GCACCGGGTAACGCTCTGGAAA	TTTTTCACTGGTCGCGCTCGGA
C308A	GCAACGCCAAATCCGCAACGTAAT	GGTGACTGCGGGCTTATCGATAT
C358A	GCAGGGCAAATTGGCGAGACG	AGGATTCGCAGCATGATCCTGTTTATC
