# Supplementary figures and images for: Cotranslational folding of alkaline phosphatase in the periplasm of Escherichia coli
Source: Protein Sci. 2020 Aug 24;29(10):2028–37. doi: 10.1002/pro.3927 (PMC7513700; doi:10.1002/pro.3927)

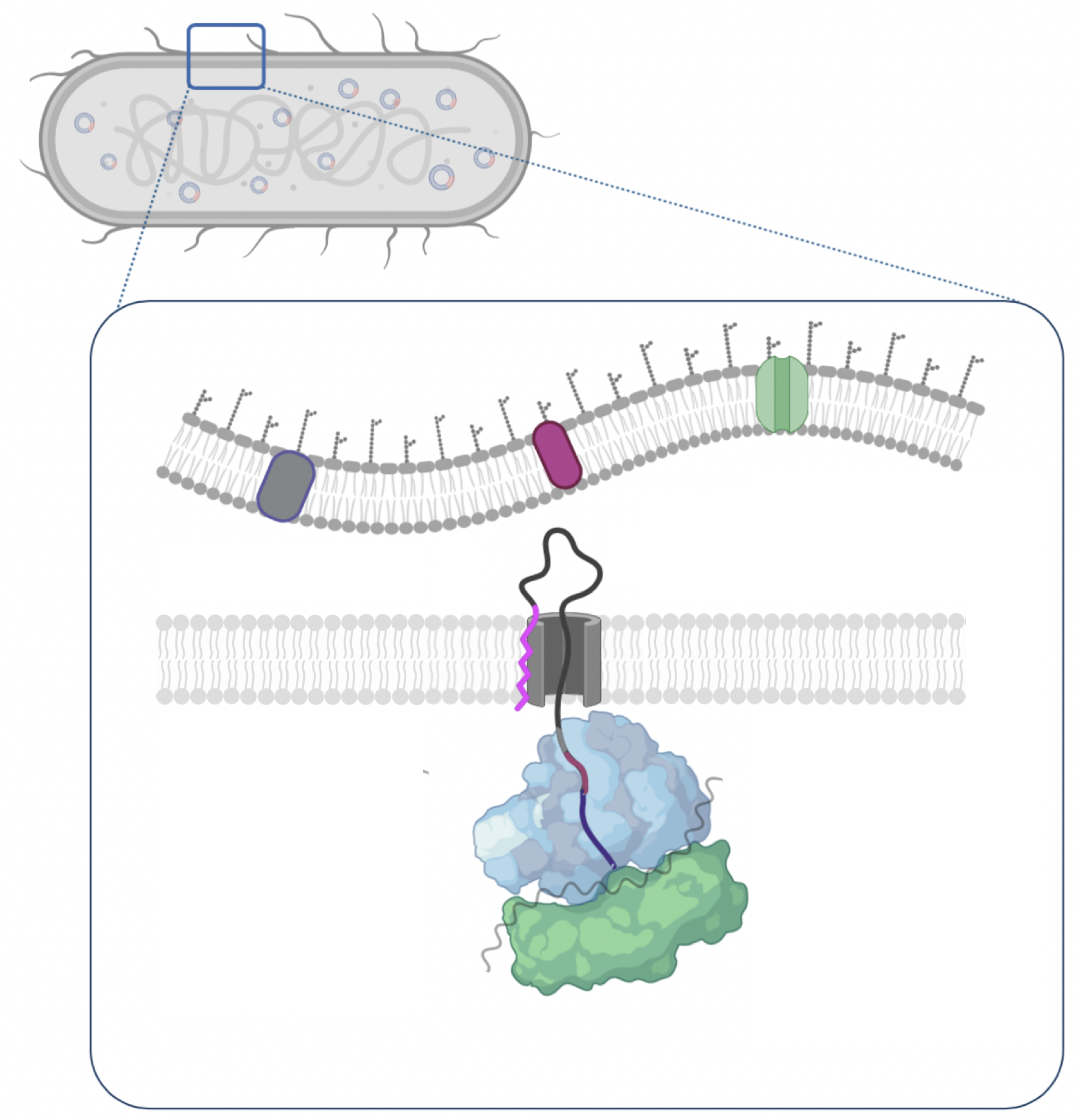

Supplement: Supplementary file 1 — APPENDIX S1: Supporting Information [file PRO-29-2028-s001.zip › PRO_3927_For consideration for use on the cover.tiff]
